# Dendritic Cell Neurofibroma With Pseudorosettes: A Case Report

**DOI:** 10.7759/cureus.90796

**Published:** 2025-08-23

**Authors:** Nabiha T Atiquzzaman, Fahad S Siddiqui, Sadia Saeed, Jacqueline Russo, Patrick Dominguez

**Affiliations:** 1 Dr. Kiran C. Patel College of Osteopathic Medicine, Nova Southeastern University, Davie, USA; 2 Dermatology, Kansas City University-Graduate Medical Education Consortium/Advanced Dermatology and Cosmetic Surgery, Maitland, USA; 3 Dermatopathology, Kansas City University-Graduate Medical Education Consortium/Advanced Dermatology and Cosmetic Surgery, Maitland, USA; 4 Pathology, OnePath Diagnostics, Jacksonville, USA

**Keywords:** dendritic cell neurofibroma with pseudorosettes (dcnp), dermato-pathology, histopathology, neural tumors differential diagnosis, pseudorosettes

## Abstract

Dendritic cell neurofibroma with pseudorosettes (DCNP) is an exceedingly rare neurofibroma variant presenting as scattered, isolated, skin-colored papules or nodules primarily on the trunk. It consists of clusters of smaller type I cells with dark nuclei and inconspicuous cytoplasm, surrounded by much larger type II cells with abundant cytoplasm and vesicular nuclei containing pseudoinclusions, resulting in the formation of distinctive pseudorosettes. While unique, it can be misdiagnosed as other neural tumors due to its histologic and clinical similarities. This report presents a unique case of DCNP in a 34-year-old male with a longstanding, asymptomatic 1.3 cm pink nodule on his mid-chest, initially suspected to be a neurofibroma. This case, along with surgical excision and histopathologic evaluation, provides valuable insight into the diagnosis of DCNP, highlighting the need for increased recognition of this rare entity and encouraging further research to refine diagnostic criteria and management strategies.

## Introduction

Initially characterized by Michal et al. in 2001, dendritic cell neurofibroma with pseudorosettes (DCNP) is a rare benign tumor of the peripheral nerve sheath [1]. DCNP is defined by two distinct cell populations: smaller “type I” cells that resemble lymphocytes, and larger “type II” cells with abundant cytoplasm, larger nuclei, and a dendritic or stellate shape. These cells form characteristic pseudorosettes, where a single type II cell is surrounded by several type I cells [2]. Pseudorosettes can mimic patterns seen in other neural tumors, such as plexiform neurofibroma or schwannoma, which may lead to misdiagnosis if not carefully evaluated.

Although the mean age of onset is around 50 years, fewer than 50 cases have been reported, underscoring the rarity of this tumor [3]. Clinically, DCNP usually presents as a solitary, skin-colored papule or nodule, most often on the trunk [1]. Its histological overlap with other peripheral nerve sheath tumors makes accurate diagnosis important, particularly to avoid misclassification and to guide appropriate immunohistochemical testing. We report a case of DCNP that initially showed histopathologic features suggestive of a neurofibroma but was ultimately confirmed as DCNP upon further evaluation.

## Case presentation

**Figure 1:**
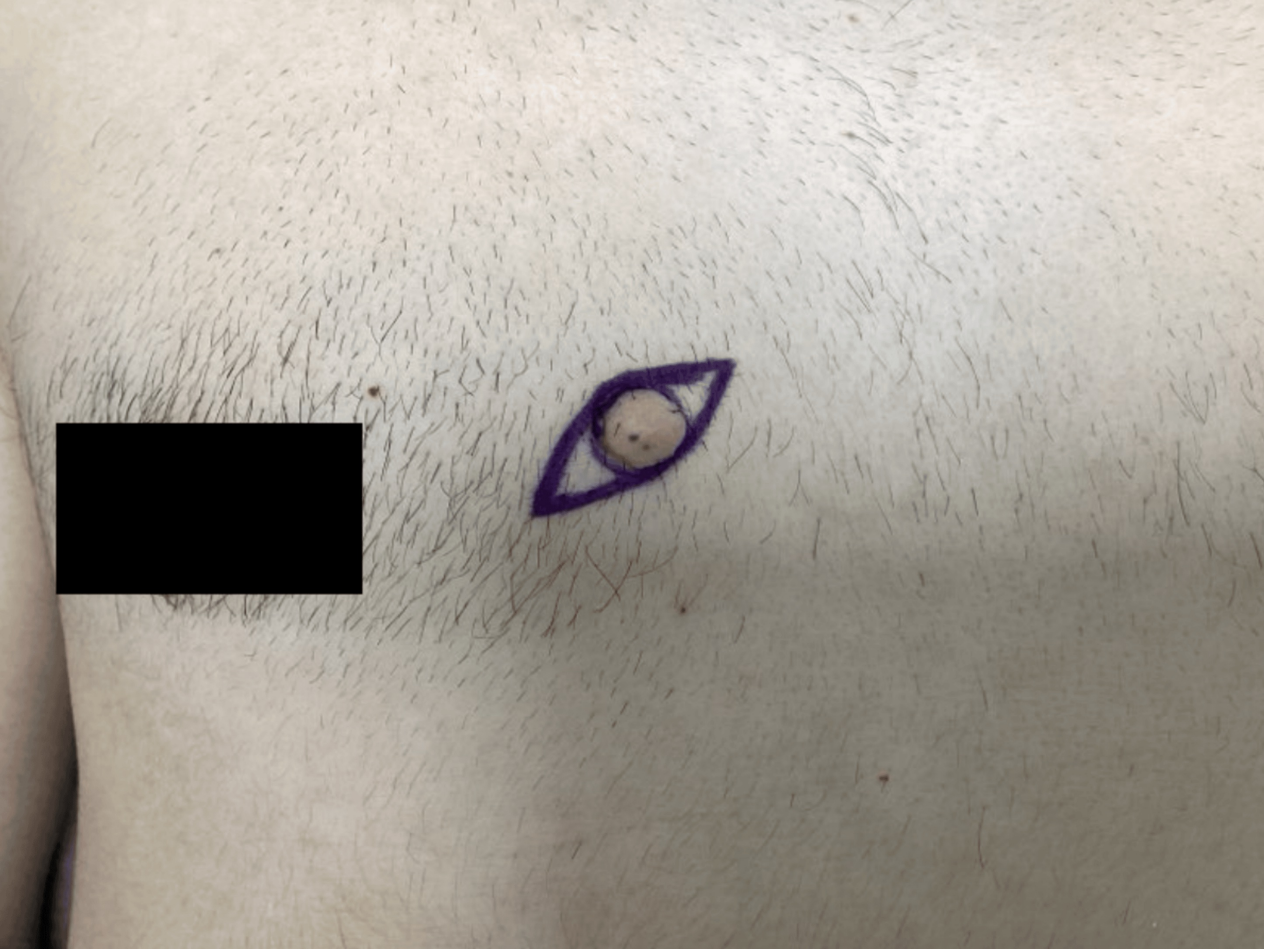
Dome-shaped pink soft nodule with a positive buttonhole sign, distributed on the left medial inferior chest and sternum

A 34-year-old male with no significant past medical history presented to the clinic with a complaint of an asymptomatic bump located on his mid-chest, which had been present for two years. On physical exam, he was found to have a 1.3 cm dome-shaped pink soft nodule with a positive buttonhole sign, distributed on the left medial inferior chest and sternum (Figure 1). The lesion was clinically consistent with a neurofibroma. The patient expressed a desire to have the lesion excised, despite being reassured of its likely benign nature. Three months later, an elliptical excision was performed, and the specimen was sent to pathology for histologic evaluation. The patient tolerated the procedure well, with minimal blood loss and no complications.

**Figure 2:**
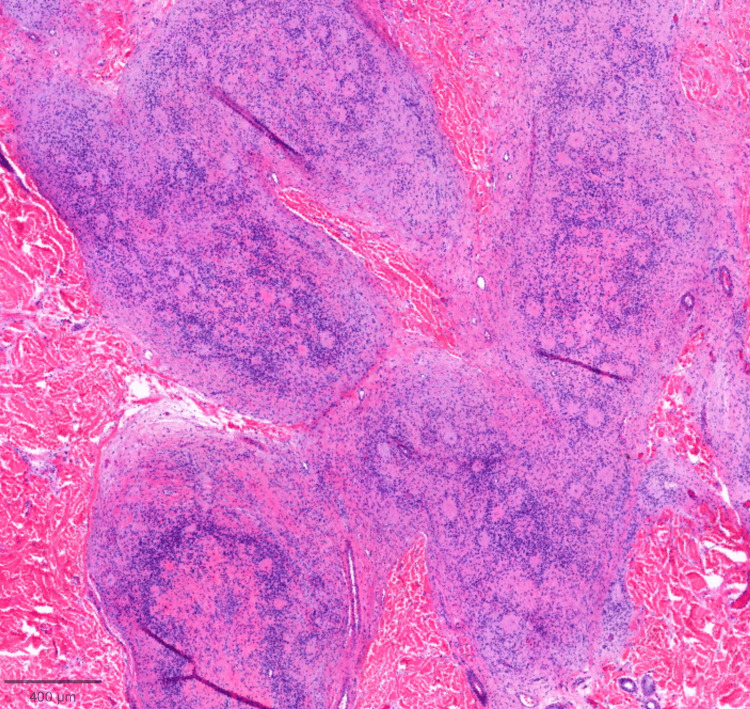
Low-power view of smaller type I cells with darker nuclei, clustered around larger type II cells with abundant cytoplasm and vesicular nuclei, forming numerous pseudorosettes

**Figure 3:**
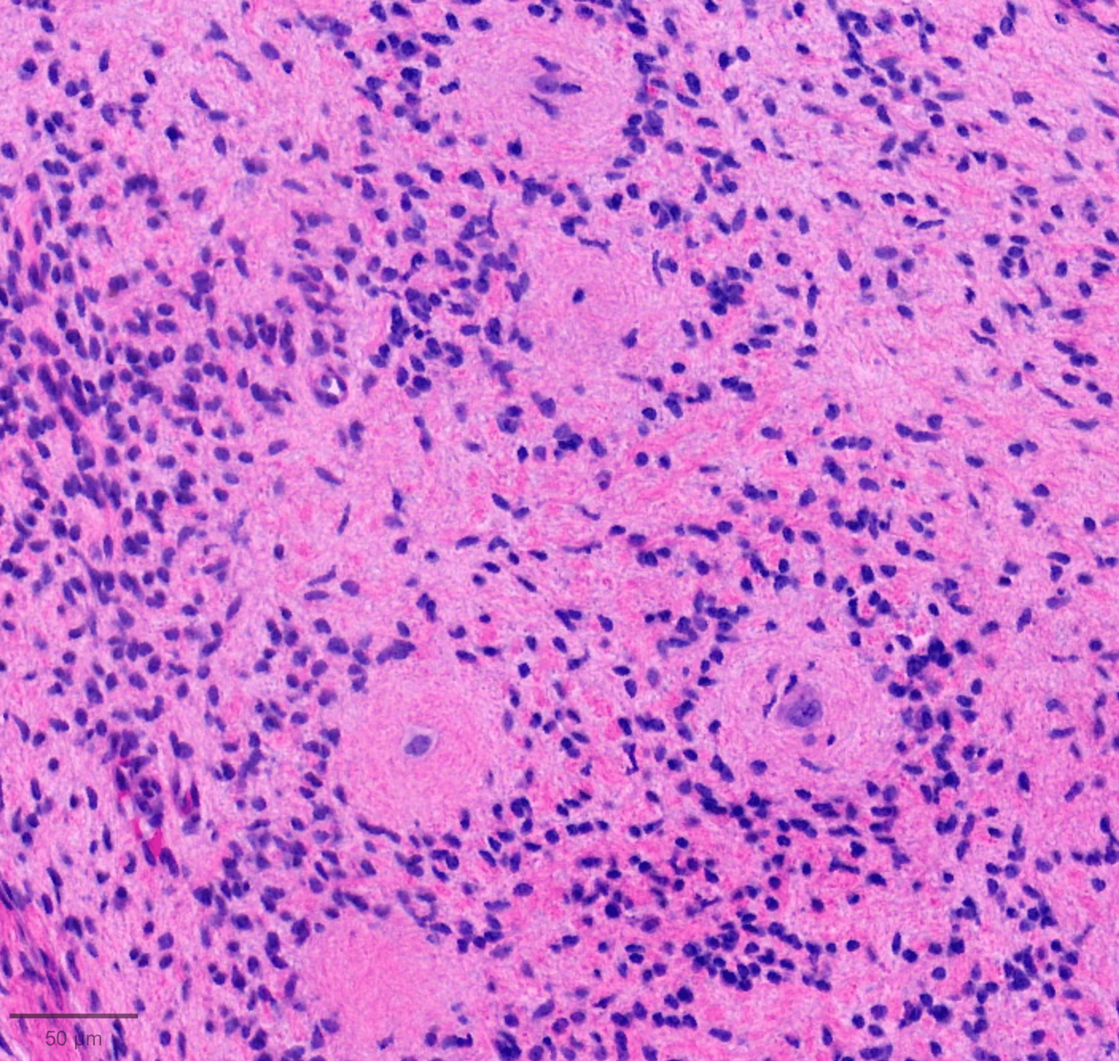
High-power view highlighting type 1 cells concentrically arranged around type II cells with eosinophilic centers, characterizing pseudorosette architecture

**Figure 4:**
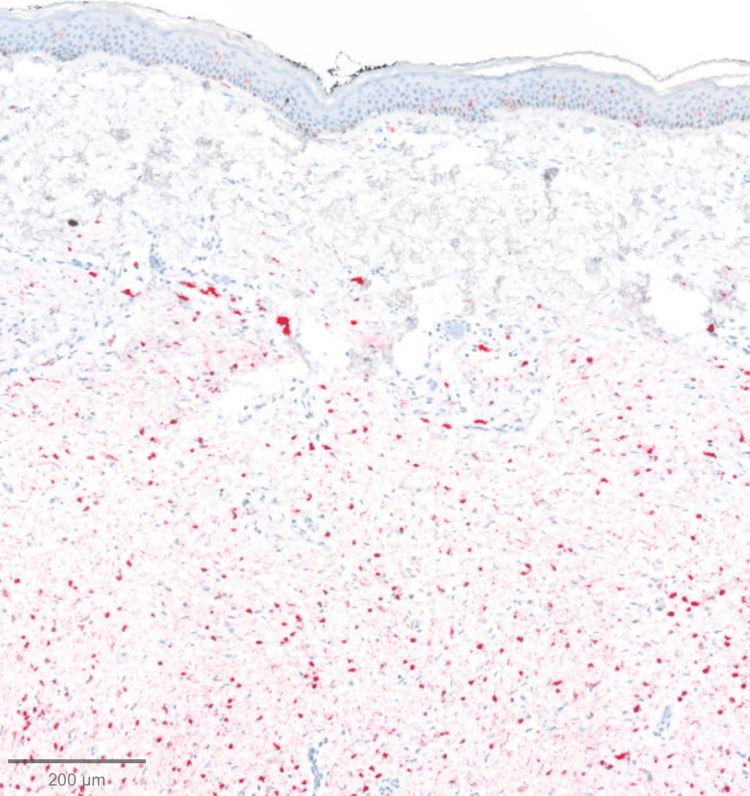
Immunohistochemical staining with diffuse S100 stain positivity

On histopathologic examination, the tumor spanned the entire reticular dermis to the level of the subcutis interface. It had a lobulated, well-defined contour. There were background delicate spindled cells with fibrillary cytoplasm, characteristic of neurofibromas. Toward the deeper aspects, the predominant pattern was of smaller type I cells with darker nuclei, clustered around larger type II cells with abundant cytoplasm and vesicular nuclei, forming numerous pseudorosettes (Figures 2-3). Lesional cells were diffusely positive for S100 (Figure 4) and SOX10. CD34 was positive in background fibroblasts. Smooth Muscle Actin (SMA), an antigen expressed by certain melanoma cells (NKI-C3), microphthalmia-associated transcription factor (MITF), and Melan-A were negative. 

Importantly, the clinical impression of a benign, slow-growing lesion correlated with the histologic findings of bland cytology, lack of atypia, and absence of mitotic activity, supporting the diagnosis of a non-syndromic DCNP. While the lesion appeared to be non-syndromic and carried a low risk of local recurrence, clinical follow-up was recommended to monitor the patient.

## Discussion

DCNP is an exceedingly rare variant of neurofibroma, with fewer than 50 cases reported in the literature since its initial description by Michal et al. in 2001 [1]. Most cases are sporadic and solitary, with no established association with neurocutaneous syndromes such as neurofibromatosis types 1 or 2. Given the rarity of this diagnosis, the exact incidence and prevalence remain unclear, and its pathogenesis is poorly understood.

There are three hypotheses about the pathogenesis of DCNP, which were outlined by Pileri et al. [4]. One possibility is that dermal interstitial dendritic cells (DIDC) from a pre-existing neurofibroma invade the lesion, though this is unlikely, as typical neurofibromas do not exhibit pseudorosette formation. Alternatively, stem cells may differentiate into Schwann-like cells and DIDCs, proliferating around peripheral nerves and giving rise to DCNP. This theory is supported by the presence of SOX10-positive cells in both type I and type II cell populations. Lastly, DIDC proliferation may be driven by trauma, inflammation, or immune responses, leading to DCNP through a hamartomatous or hyperplastic mechanism.

The histopathological qualities of DCNP can be comparable to plexiform neurofibroma (PNF) and neuroblastoma-like schwannoma. PNFs are peripheral nerve tumors caused by bi-allelic loss of NF1 in the Schwann cell (SC) lineage [5]. They present as bag-like masses within the skin and its multifocal tumor nodules. Histologically, there are no pseudorosettes present in PNF. However, a neuroblastoma-like schwannoma shows histological similarities with DCNP, exhibiting rosette-like structures and presenting clinically as subcutaneous nodules [6]. DCNP stains positive for S100 and SOX10, and similarly, a majority of schwannomas and neurofibromas show a relatively increased expression of both these markers [7].

DCNP carries specific management implications due to its rarity and distinct histopathological features. While surgical excision with clear margins is generally curative, ongoing follow-up is recommended to monitor for local recurrence. The lack of significant atypia or mitotic activity in most cases supports its benign nature, but it is vital to stay vigilant in the case of a recurrence or misdiagnosis. Patients diagnosed with DCNP should be educated about its benign nature and the low risk of recurrence, as in this case presented. Periodic dermatologic evaluations may be prudent to ensure that no additional lesions or changes occur at the surgical site. In the case of recurrence, it is recommended that there be prompt suspicion, excision, and pathological examination.

## Conclusions

This report contributes to the limited body of documented cases of DCNP, a rare variant of neurofibroma with distinct histological and immunohistochemical features. While its clinical course is benign, this case highlights the critical need for increased diagnostic vigilance to avoid misclassification, particularly given its overlap with other neural tumors. It illustrates the importance of integrating clinical presentation with detailed histologic architecture and immunoprofiling to achieve accurate diagnosis. Given the rarity of DCNP, we advocate for documentation of similar cases as well as long-term follow-up studies to better characterize its natural history, pathogenesis, and management.
